# Ancient Antimicrobial Peptides Kill Antibiotic-Resistant Pathogens: Australian Mammals Provide New Options

**DOI:** 10.1371/journal.pone.0024030

**Published:** 2011-08-30

**Authors:** Jianghui Wang, Emily S. W. Wong, Jane C. Whitley, Jian Li, Jessica M. Stringer, Kirsty R. Short, Marilyn B. Renfree, Katherine Belov, Benjamin G. Cocks

**Affiliations:** 1 Biosciences Research Division, Department of Primary Industries, Bundoora, Australia; 2 Faculty of Veterinary Sciences, University of Sydney, Sydney, Australia; 3 Australian Research Council Centre of Excellence in Kangaroo Genomics, Parkville, Australia; 4 Monash Institute of Pharmaceutical Sciences, Monash University, Parkville, Australia; 5 Department of Zoology, The University of Melbourne, Parkville, Australia; 6 Department of Microbiology and Immunology, The University of Melbourne, Parkville, Australia; 7 La Trobe University, Bundoora, Australia; Ludwig-Maximilian-University, Germany

## Abstract

**Background:**

To overcome the increasing resistance of pathogens to existing antibiotics the 10×^'^20 Initiative declared the urgent need for a global commitment to develop 10 new antimicrobial drugs by the year 2020. Naturally occurring animal antibiotics are an obvious place to start. The recently sequenced genomes of mammals that are divergent from human and mouse, including the tammar wallaby and the platypus, provide an opportunity to discover novel antimicrobials. Marsupials and monotremes are ideal potential sources of new antimicrobials because they give birth to underdeveloped immunologically naïve young that develop outside the sterile confines of a uterus in harsh pathogen-laden environments. While their adaptive immune system develops innate immune factors produced either by the mother or by the young must play a key role in protecting the immune-compromised young. In this study we focus on the cathelicidins, a key family of antimicrobial peptide genes.

**Principal Finding:**

We identified 14 cathelicidin genes in the tammar wallaby genome and 8 in the platypus genome. The tammar genes were expressed in the mammary gland during early lactation before the adaptive immune system of the young develops, as well as in the skin of the pouch young. Both platypus and tammar peptides were effective in killing a broad range of bacterial pathogens. One potent peptide, expressed in the early stages of tammar lactation, effectively killed multidrug-resistant clinical isolates of *Pseudomonas aeruginosa, Klebsiella pneumoniae and Acinetobacter baumannii.*

**Conclusions and Significance:**

Marsupial and monotreme young are protected by antimicrobial peptides that are potent, broad spectrum and salt resistant. The genomes of our distant relatives may hold the key for the development of novel drugs to combat multidrug-resistant pathogens.

## Introduction

Over the past two decades a large number of antimicrobial peptides have been identified in plants, animals and microorganisms. These endogenous antibiotic peptides play a key role in the innate immune system and are a first line of defense in protecting the internal and external body surfaces of the host. The best known antimicrobial peptide gene families include the defensins and the cathelicidins. In this paper we focus on the cathelicidin gene family. Cathelicidin genes are characterized by a conserved signal sequence and pro-peptide region but are highly variable in the C-terminal domain that encodes the mature antimicrobial peptide, which is released by elastase cleavage. The active peptides vary in length both within a species and between species and range from 12–100 amino-acid residues [1], [2]. Cathelicidins are found in neutrophils and macrophages, as well as epithelial cells of the testis, skin, gastrointestinal tract and respiratory tract (reviewed in [2]). Cathelicidins interact with and destroy Gram-positive and Gram-negative bacteria, protozoa and fungi via electrostatic interactions between their positively charged peptides and the negatively charged molecules found in the cell membranes of their targets. Besides their direct antimicrobial function, cathelicidins also play a role in inflammation and in dampening of excessive inflammation, cell proliferation and migration, immune modulation, wound healing, angiogenesis and the release of cytokines and histamine (reviewed in [2], [3]).

The best studied cathelicidin is human LL-37, the only human cathelicidin. It has anti-tumour and anti-HIV activity [4]. Recurrent bacterial infections occur in patients with Chediak-Higaski syndrome, where mature neutrophils lack elastase to cleave cathelicin pro-peptides [5]. Cathelicidin knockout mice are susceptible to Group A *Streptococccus*, herpes simplex virus, Escherichia coli and vaccinia virus (reviewed in [6]). In the rat, cathelicidins protect against lethal sepsis caused by Gram-negative bacteria [7].

During the course of evolution, nature has developed a vast array of antimicrobial peptides. Each species contains a different set of related genes that reveal signatures of different selective forces. Cathelicidin genes have been identified in the invertebrate hagfish [8] and in a range of vertebrates, including mammals [9], [10], [11], chickens [12], [13], [14], fish [15], [16] and reptiles [17], [18], [19], [20].

Marsupials and monotremes hold an important position in the vertebrate phylogenetic tree because they represent two of the three extant mammalian lineages. Marsupials and eutherian mammals last shared a common ancestor between 130 and 148 million years ago, while monotremes and therian mammals (marsupials and eutherians) diverged about 166 million years ago [21], [22], [23]. Marsupials and monotremes differ from eutherian mammals primarily in their mode of reproduction. Marsupials give birth to underdeveloped (altricial) young after a short gestation, which in the tammar wallaby (*Macropus eugenii*) is only 26.5 days [24]. At birth, the neonate weighs only 440mg and is 16–17mm long [24]. This developmental stage is roughly equivalent to a 40 day human embryo or a 15 day mouse embryo. The young remains in the pouch for 9–10 months, supported by a long and physiologically sophisticated lactation. Initially the young are permanently attached to a teat, but later they begin to release the teat periodically whilst still in the pouch. The mother's milk undergoes compositional changes over time ensuring that the nutritional supply to the young is specifically matched to each developmental stage [25]. At birth the wallaby does not have a differentiated immune system. Cells involved in adaptive immunity are not seen until about 35 days after birth [26], [27] and immunocompetence develops around 90–100 post partum [28].

Monotremes lay eggs and the young hatch at a very early stage in development similar to that of the marsupial neonate. The platypus (*Ornithorhynchus anatinus*) lays up to 3 leathery-shelled eggs ∼15–21 days after mating [29]. After a 10 day incubation period, young approximately 15mm in length emerge [30]. Much of the development, including that of the immune system, occurs before weaning. During the three to four months before they leave the burrow the young grow from ∼1.5 cm to ∼40 cm [30]. Platypuses do not have teats but the milk is secreted via mammary patches on the female's abdomen. It is not known exactly when platypus immune tissues reach maturity, but by the time platypuses are adults, they possess a range of lymphoid tissues that are histologically similar to those of therian mammals [31].

Unlike eutherians, whose immune systems develop in the relatively sterile confines of the mother's uterus, the marsupial and monotreme immune systems develop while exposed to a range of pathogens in the pouch and in the burrow. In eutherians, cathelicidins play a crucial role in neonatal defense. They have been detected in skin of neonatal mice and humans, in human milk, and LL-37 is found in the vernix caseosa, a creamy substance that covers the healthy infant at birth (reviewed in [32]). Therefore it is likely that the marsupial and monotreme genomes have also evolved under evolutionary pressure to protect immunologically naïve young with broad spectrum antibiotics and that their genomes will reveal a source of novel antimicrobial peptides that may provide unique answers to antibiotic resistance.

The recent sequencing of the tammar wallaby genome [33] and the platypus genome [23] allow discovery of these divergent peptides using bioinformatics strategies. There are seven cathelicidin genes in the tammar mammary gland EST library [32], expressed in adult leukocytes and in primary and secondary lymphoid organs of the pouch young over the first 120 days of life. An eighth cathelicidin, recently identified, is expressed in spleen and gastrointestinal tract of newborn animals and in most tissues by seven days after birth [34]. We identified eight cathelicidin genes in the platypus genome [23]. All were expressed in adult brain, kidney, liver, lung, spleen and testis [35]. Surprisingly, none of them were expressed in the single platypus milk sample available to us [35]. It is important to note that access to platypus samples for scientific research is difficult. In the wild, adults are trapped in nets without their young, as the young remain in the burrow while the mother forages. Therefore, it is impossible to know the stage of the mother's lactation. It is possible that milk samples from other stages of lactation could have yielded a different result. Due to ethical and conservation concerns, further study of monotreme developmental immunology is not possible at this time and awaits opportunistic sampling.

In this paper we describe functional testing of antimicrobial peptides identified through genome data-mining and support this with expression studies that confirm that marsupials (and probably monotremes) use these powerful broad-spectrum peptides to protect themselves and their young from a diverse range of pathogens found in the pouch (and in the soil).

## Results and Discussion

### Identification of cathelicidin genes

**Figure 1:**
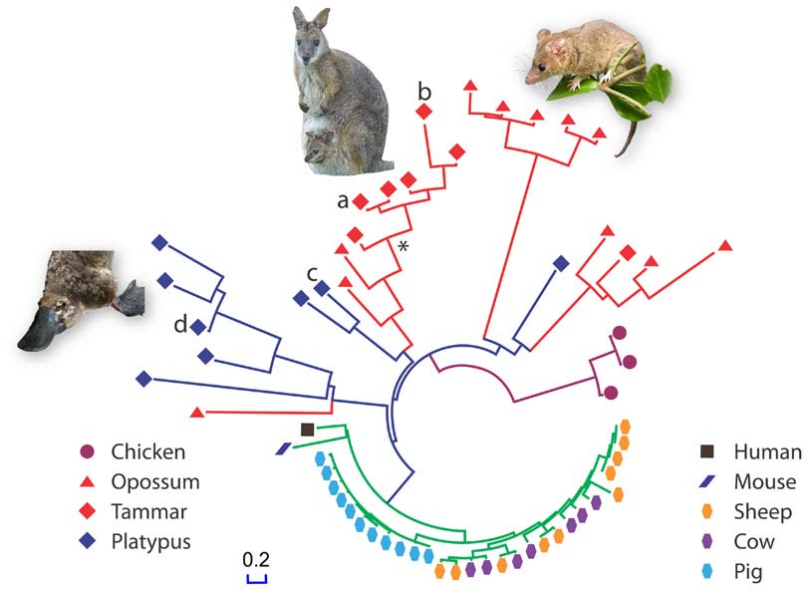
Phylogenetic tree demonstrating superior diversity of antimicrobial peptides in non-eutherian mammals. Tammar cathelicidin peptides. WAM1 (a) – KRGFGKKLRKRLKKFRNSIKKRLKNFNVVIPIPLPG from MaeuCath1 (Genbank EF624481.1), WAM2 (b) -KRGLWESLKRKATKLGDDIRNTLRNFKIKFPVPRQG from MaeuCath5 (Genbank EF624484.1), Ancestral WAM (*)- RRGFWKRLRRRLRRFGDRIRNRFRNFREKLPDPFPG. Platypus cathelicidin peptides PAM1 (c) – RTKRRIKLIKNGVKKVKDILKNNNIIILPGSNEK from OranCath1 [23] and PAM2 (d) – RPWAGNGSVHRYTVLSPRLKTQ from OranCath2 [23].

Fourteen divergent cathelicidin genes were identified in the tammar genome, twelve in the opossum and eight in the platypus (Fig. 1). Sequences were identified using a PFAM hidden Markov model and full-length coding sequences were extracted by gene prediction. The phylogenetic relationships of cathelicidins are shown in Fig. 1. Proteins selected for active C-terminal peptide prediction and testing are indicated.

### Expression of cathelicidin genes

**Figure 2:**
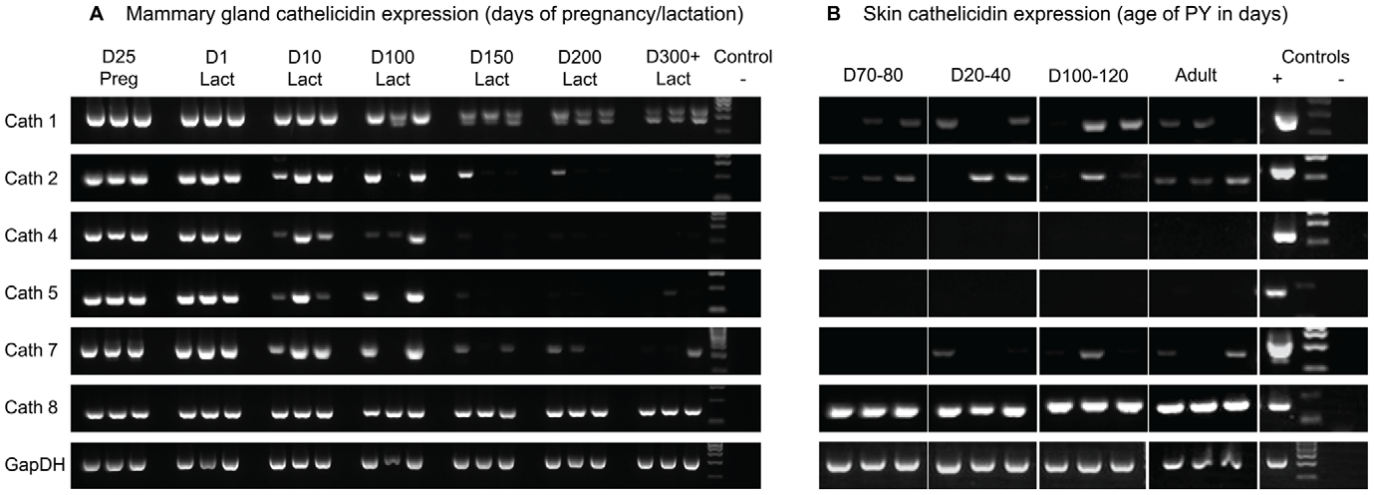
Tammar cathelicidin gene expression in the mammary gland throughout lactation and in pouch young skin. D = Day; PY = pouch young. Cath1 and Cath5 correspond to WAM1 and WAM2, respectively. (a) Tammar cathelicidin gene expression in the mammary gland throughout the 350 days of lactation. (b) Tammar cathelicidin gene expression in male D20–D120 pouch young skin and adult lactating female pouch skin. Female pouch young skin and adult non-lactating pouch skin showed similar expression patterns (not shown). Each age group is represented by three different samples and mammary gland and thymus tissues were used as positive controls (+). No template controls (–) were clean.

Tammar cathelicidin genes were expressed in the mammary gland throughout lactation, consistent with a role in protection of immuno-naïve young. Five out of six genes tested were down-regulated after 100 days post partum (Fig. 2a) corresponding to the time when the young achieve immune competence. Similar cathelicidin expression also occurred in the skin of pouch young and adult in four of the six genes tested (Fig. 2b).

### Evolution of cathelicidin genes

Marsupial and monotreme antimicrobial peptides are ancient, diverging from each other approximately 211 million years ago (101–340 million years, 95% credibility interval (CI)). Mammalian ancestors likely expressed a broad repertoire of cathelicidin peptides to protect their young. Over time, with the evolution of longer gestation periods and the birth of young with greater immune competence, cathelicidin genes were lost from the eutherian lineage, because humans and mice have only one cathelicidin gene. Recent gene duplications in sheep, cows and pigs, ∼53 MYA (27–80, 95% CI) (Fig. 1) were likely driven by increased pathogen pressures in herd animals.

The high level of sequence divergence of the marsupial and monotreme cathelicidin genes (tammar peptides share ∼28% amino-acid identity with each other) suggests that they should have the potential to inhibit a wide range of microbial pathogens.

### Activity of tammar and platypus cathelicidin genes

We selected four divergent tammar (wallaby antimicrobial 1 and 2:WAM1 and 2) and platypus antimicrobial 1 and 2 (PAM1 and PAM2) peptides to synthesize and test (Fig 1). All four killed the Gram-positive bacteria *Bacillus subtilis, Staphylococcus aureus, Streptococcus uberis* and *Strep. pyogenes*, and the Gram-negative bacteria *Escherichia coli*, *Salmonella choleraesuis* and *P. aeruginosa* (Table 1). PAM2, WAM1 and WAM2 also killed the fungal pathogen *Candida albicans* (Table 1). Our four divergent peptides were more potent than the human cathelicidin peptide LL-37 which was less effective against the seven bacteria tested and did not kill *C. albicans*. The platypus peptides had distinct activity profiles, with PAM2 active against *C. albicans* and PAM1 more potent against *S. aureus*

**Table 1:** Antimicrobial activities of cathelicidin peptides.

Strains	MIC (µM)					
	WAM1	WAM2	Ancestral WAM	LL37	PAM1	PAM2
**Gram-negative**						
*E. coli* DH5α	0.47	1.46	0.41	5.57	0.75	0.87
*Sal. enterica* (ATCC 14028)	1.14	1.58	0.96	4.30	0.47	1.11
*P. aeruginosa* (ATCC 27853)	0.77	1.29	2.06	>56	1.89	0.73
**Gram-positive**						
*B. subtilis*	1.50	2.14	1.56	8.62	1.96	3.37
*S. aureus* (ATCC 25923)	1.01	1.39	1.42	3.01	0.56	2.42
*Strep. pyogenes* (ATCC 19615)	0.66	0.39	0.68	>55	0.73	0.83
*Strep. uberis*	1.22	0.63	0.07	2.24	0.25	0.51
**Fungi**						
*C. albicans* (ATCC 753)	1.30	1.47	6.45	>56	>20	2.50

### A phylogenetic approach to designing additional antimicrobial peptides

A phylogenetic approach was used to design additional antimicrobial peptides. An ancestral peptide (WAM =  RRGFWKRLRRRLRRFGDRIRNRFRNFREKLPDPFPG) was designed using MaeuCath1, 3, 5, 6 and 7 and three methods: PAML [36], GASP [37] and Ancescon [38]. 6/40 amino acid positions in the putative ancestral peptide were ambiguous and in these positions the Ancescon predicted residues were selected. We predict this peptide originated ∼59 MYA (Fig. 1) and is ancestral to the major clade of marsupial antimicrobial peptides including the modern active peptides WAM1 and WAM2. This ancestral peptide from the Paleocene had broad-spectrum activity and was approximately ten times more effective than LL-37 at killing *E. coli*, and *P. aeruginosa*. The archaic peptide had particularly potent activity against the mastitis pathogens *Strep*. *pyogenes* and *Strep. uberis* (Table 1), suggesting that an ancestral function included mammary gland protection. This approach provides a new avenue to investigate ancient peptides and their immune functions.

### WAM1- highly potent but not toxic

**Figure 3:**
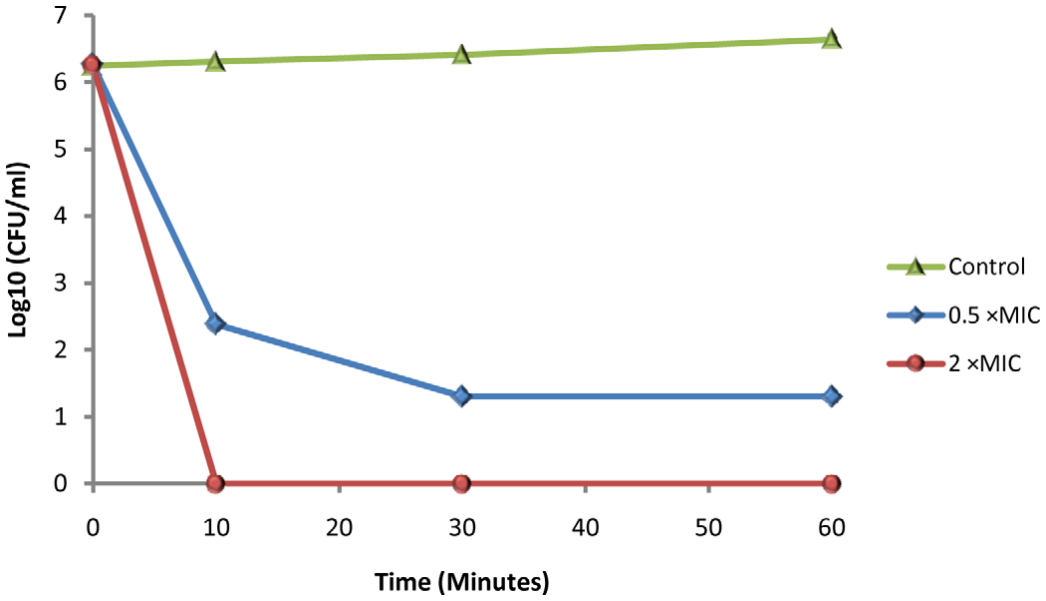
Time course of *K. pneumoniae* killing by WAM1 in broth. Time marked in minutes. [MIC]  =  0.47 µM. Antimicrobial assays were performed as described previously [57].

**Figure 4:**
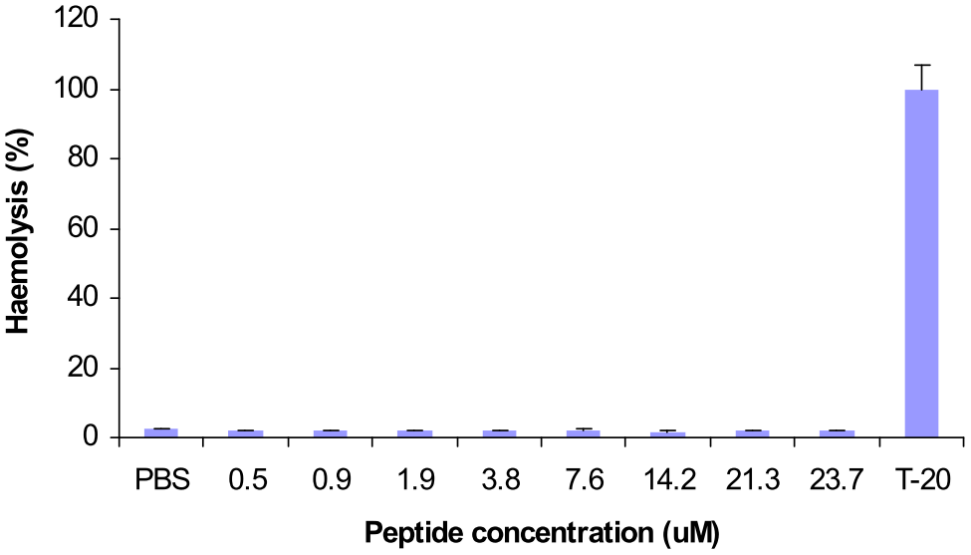
Toxicity of WAM1 on human red blood cells. Haemolytic activity was determined by treating human red blood cells with different concentrations of WAM1 and haemoglobin release measured by absorbance at 450 nm. Samples were incubated at 37°C for 1 hour. PBS and 1% Tween-20 were used as negative and positive controls, representing 0% and 100% hemolytic activity respectively.

The tammar peptide WAM1 was found to be remarkably potent. It is 3–80 times more effective than LL-37 against each of these microbes and is ten times more effective against *E. coli* and *B. subtilis* than antibiotics such as ampicillin, tetracycline and chloramphenicol (Table 2). WAM1 was selected for further studies (results summarized in Table 3) as it was effective against Gram-negative bacteria including *P. aeruginosa*, a bacterial species known to cause untreatable infections due to its resistance to all current antibiotics. We also tested nineteen clinical isolates of multidrug-resistant microorganisms that are resistant to at least three antibiotics. WAM1 killed multidrug-resistant gram-negative bacteria including *P. aeruginosa, A. baumannii* and *K. pneumoniae* (Table 3). This is significant as some of the *P. aeruginosa* isolates are also resistant to the last-line therapy, colistin [39]. Unlike some other antimicrobial peptides, eg LL37 [40], WAM1 is resistant to inhibition by high (150–200 mM) NaCl concentrations (Table 4), which is relevant as most alpha-helix antimicrobial peptides lose activity at high salt concentrations. The salt resistance of WAM1 suggests that it may be suitable for applications *in vivo* [41], [42], [43], [44]. WAM1 was rapidly bactericidial, killing 99.9% of K. *pneumoniae* within 15 minutes (Fig. 3). WAM1 was not hemolytic against human red blood cells indicating potential for parenteral use in humans (Fig. 4). The effectiveness of tammar cathelicidin WAM1 in killing multidrug-resistant *P. aeruginosa* demonstrates the potential of these compounds for treating life-threatening drug resistant infections.

**Table 2:** Comparison of WAM1 potency to antibiotics ampicillin, tetracycline and chloramphenicol against *E. coli*.

	MIC (µM)	Relative potency of WAM1
**Ampicillin**	5.7	12 x
**Tetracycline**	4.5	10 x
**Chloramphenicol**	12.4	26 x
**WAM1**	0.47	

**Table 3:** Activity of WAM1 against antibiotic-resistant gram-negative clinical isolates.

Isolate description	MIC (µM)
*P. aeruginosa* (ATCC 27853)	0.95
*P. aeruginosa* 001*	1.90
*P. aeruginosa* 002*	0.95
*P. aeruginosa* 003*	0.47
*P. aeruginosa* 004*	1.90
*P. aeruginosa* 005* (colistin R)	0.95
*P. aeruginosa* 006* (colistin R)	0.95
*P. aeruginosa* 007* (colistin R)	0.95
*P. aeruginosa* 008* (colistin R)	0.95
*P. aeruginosa* 009* (colistin R)	0.95
*P. aeruginosa* 010* (colistin R)	>30.4
*P. aeruginosa* 011*	0.47
*P. aeruginosa* 012*	1.90
*P. aeruginosa* 013*	>30.4
*P. aeruginosa* 014*	>30.4
*A. baumannii* (ATCC 19606)	1.90
*A. baumannii* 001*	0.95
*A. baumannii* 002*	>15.2
*A. baumannii* 003*	1.90
*K. pneumoniae* (ATCC 13883)	0.47
*K. pneumoniae* 001*	1.90
*K. pneumoniae* 002*	0.95
*K. pneumoniae* 003*	7.59

The 19 antibiotic-resistant isolates are marked with an asterisk and those also resistant to colistin are indicated.

**Table 4:** WAM1 activity in the presence of high concentrations of NaCl.

	MIC (µM)0 mM NaCl	MIC (µM)150 mM NaCl	MIC (µM)200 mM NaCl
*P. aeruginosa*ATCC 27853	0.9	0.9	0.9
*A. baumannii*ATCC 19606	1.9	1.9	3.8
*K. pneumoniae*ATCC 13883	0.5	0.9	0.9

Our data suggests that the function of WAM1 is to protect the altritical neonate at birth, as clinically relevant bacteria affected by WAM1 (such as *A. baumanni* and *E. coli*) were detected in the tammar pouch around the time of birth (Table 5). Consistent with previous reports [45], we also identified a number of potentially novel bacterial species in the pouch. This may indicate that WAM1 is able to limit the growth of a broader spectrum of bacterial species, beyond those tested in this study.

**Table 5:** Bacteria identified by 16S rDNA sequencing from the pouch of the tammar wallaby around the time of birth.

Time of colony isolation	16S rDNA identification	Percentage match
Day(-6)	*Bacillus licheniformis*	100%
Day(-5)	*Bacillus licheniformis*	100%
Day(-5)	*Devriesea agamarum* a.	94%
Day(-5)	*Staphylococcus equorum*	99%
Day(-2)	*Corynebacterium tuscaniense* a.	95%
Day(-2)	*Bacillus cereus*	99%
Day(0)	*Escherichia coli*	100%
Day(0)	*Acinetobacter baumannii*	100%
Day(0)	*Corynebacterium urealyticum*	98%
Day(0)	*Enterobacter aerogenes*	100%
Day(0)	*Corynebacterium coyleae* a.	95%
Day(0)	*Devriesea agamarum* a.	95%
Day(+1)	*Devriesea agamarum* a.	95%
Day(+1)	*Escherichia coli*	100%
Day(+1)	*Acinetobacter baumannii*	100%

a. The low sequence homology observed in these isolates suggests that they may be a novel bacterial species and/or genus. Day (-6, 5, 2) etc indicates 6, 5 and 2 days before birth; Day(0) is day of birth; Day(+1)is the day after birth.

### Conclusions

The dwindling numbers of therapeutic options for bacterial ‘superbugs’ necessitates the development of novel antimicrobials. The development of microbial resistance against naturally occurring antimicrobials peptides is rare [2], and our data suggest that the potent antimicrobial peptide gene expansions in the platypus and tammar genomes are good candidates for discovering natural peptides to fight multidrug resistant microbes. The strong antimicrobial activity, salt insensitivity and low haemolytic activity further suggest that marsupial and monotreme cathelicidin peptides have the potential to provide novel therapeutic antibiotics.

## Materials and Methods

### Identification of tammar and platypus cathelicidin genes

#### Genomic search

The PFAM cathelicidin HMMER [46] profile (PF00666) was used to search a six-frame translated assembly of the tammar wallaby genome (1.0). Gene predictions were performed using FGENESH+ [47] based on HMMER results. These were confirmed by BLAST [48] against NCBI's protein nr database. Platypus and tammar cathelicidin sequences can be downloaded from http://hp580.angis.org.au/tagbase/gutentag/. GenBank Accessions for previously published cathlicidin sequences [32], [34] are MaeuCath1 EF624481.1, MaeuCath2 EF624482.1, MaeuCath3 EF624483.1, MaeuCath4 EF624484.1, MaeuCath5 EF624485, MaeuCath6 EF624486.1, MaeuCath7 EF624487.1, MaeuCath8 EU883635.1.

#### Gene expression of cathelicidin genes

All experiments were approved by the University of Melbourne Animal Experimentation Ethics Committee and the animal handling and husbandry procedures were in accordance with the National Health and Medical Research Council of Australia (2004) guidelines. Pregnancy was initiated in females carrying an embryo in diapause by the removal of their pouch young (RPY) [49], [50]. Adult females carrying fetuses in the final third of gestation (day 19–26/birth) or pouch young (day 0–350) were euthanised either by cervical dislocation or by an anaesthetic overdose (sodium pentobarbitone, 60 mg/ml, to effect) and portions of the suckled mammary gland and pouch skin were collected and snap frozen in liquid nitrogen. Pouch young (PY) skin also was collected from both male and female animals of various ages. Tissues were homogenized and total RNA was extracted from mammary glands using RNeasy Lipid Tissue Minni Kit (QIAGEN, #74804) or from pouch and PY skin using Tri-Reagent (Ambion #AM9738) as described by the manufacturer with a final elution of RNA in 60–80 µl of RNAsecure H_2_O (Ambion, Geneworks, # AM7005) in a dilution of 1/24 µl. Total RNA was DNase treated (DNA-freeTM, Ambion, # 1906) to remove contaminating genomic DNA, quantified with a nano-spectrometer (NanaDrop ND-1000 Spectrophotometer, NanoDrop Technologies Inc, Wilmington, DE, USA) and cDNA was synthesized with SuperScript III First Strand Synthesis System for RT-PCR (Invitrogen, # 11904-018). Typically 2000 ng or a maximum of 8 µl of total RNA was used in each cDNA synthesis reaction, with 1 µl of Oligo (dT)12–18 (50 µM). cDNA integrity was immediately assessed with *GAPDH* RT-PCR (Table 1). Approximately 0.5–1 µl (50–100 ng) of template was used with 0.5 µM of each primer (table 1) with GoTaq Green Master Mix (PROMEGA, # M7122) RT-PCR cycles consisted of 94°C for 1 min, followed by 35 cycles of 15 sec at 94°C, 30 sec at 60–63°C, and 40 sec at 72°C, and a final extension at 72°C for 5 min. PCR products were resolved by gel electrophoreses and a band of each cathelicidin was extracted (QIAquick Gel Extraction Kit, Qiagen, # 28704) for direct sequencing. Sequences were assessed with FinchTV (v.1.3.1) DNA sequence chromatogram trace viewer software to confirm correct amplification. Cathelicidin expression RT-PCRs were performed using gene specific primers (Table 6). Three separate samples from each age group were used to assess the expression patterns in the mammary gland throughout lactation and in the skin throughout development. All primers were designed using Primer3 (v. 0.4.0) [51] and synthesised by Sigma-Aldrich.

**Table 6:** Primer sequences for cathelicidin sequence expression analysis.

Cathelicidin	Primer	Primer sequence (5′ to 3′)	Length (bp)
3	Fw	AGTGGGTGAAAAAGTTAAGACCAG	198
	Rv	TATGAGAAGAAGGGTGAGGGTAAG	
1,2,4,5,6,7	Fw	CCATACCAGGATGTGCTGAAT	
1	Rv	ACAGGAGGCTACCCTGGCAGT	413
2	Rv	AGTCAGAATCCCTTCCCAGCC	245
4	Rv	AGTCAGACTCCCTCCCTAGTC	242
5	Fw	CATGCAGGTACTCCTATTGGTGCTG	190
	Rv	AATGCATTGTTTCACCAGCTCCTC	
7	Rv	ATCATCCCCGAGTTTCGTCAC	345
8	Fw	ATCTACTCTCCTTCACCCAATCAG	126
	Rv	GGATACTGAGCCTTGACATTCTTT	
GAPDH	Fw	CCTACTCCCAATGTATCTGTTGTGG	351
	Rv	GGTGGAACTCCTTTTTTGACTGG	

### Construction of phylogenetic tree shown and divergence dating

The phylogenetic tree was constructed using the prepro region (cathelin domain) of the peptide sequences using the neighbor-joining method with Jones-Taylor-Thornton amino-acid substitution as implemented by MEGA4 [52]. Amino-acid substitution model was identified with ProtTest [53] using Bayesian information criterion. We used partial deletion and gamma distribution for variation among sites (shape parameter = 1.0) in MEGA4 [52]. Divergence dating was performed using BEAST v1.5. Tree prior was defined by Yule process. The BLOSUM 62 amino-acid substitution model was used. We used an uncorrelated log-normal relaxed-clock model to account for lineage-specific heterogeneity. A log-normal calibration node defined at boreoeutheria divergence [54]. Priors for each parameter followed a normal distribution with a standard deviation of 0.5 million years. Monte Carlo stimulations were run for 10,000,000 steps and 10,000 trees were stored and 9,000 trees summarized. Accession numbers: tammar1 - ABV01938.1, tammar2 - ABV01939.1, tammar3 - ABV01940.1, tammar4 - EF624484.1, tammar5 - ABV01941.1, tammar6 - ABV01942.1, tammar7 - ABV01943.1, tammar8 - ACJ76797.1, human - NP_004336.2, mouse - AAB88303.1, chicken1 - NP_001001605.1, chicken2 -Q2IAL7.1, chicken3 - AAZ65841.1, cow1 - P22226, cow2 - P19660, cow3 - P19661, cow4 - P33046, cow5 - P54229, cow6 - P54228, cow7 - P56425, sheep1 - P54230, sheep2 - P82018, sheep3 - P79362, sheep4 - P50415, sheep5 - P49928, sheep6 - P49929, sheep7 - P79361, sheep8 - O19031, sheep9 - O19040, sheep10 - P79360, pig1 - P15175, pig2 - P51524, pig3 - P32194, pig4 - P32195, pig5 - P32196, pig6 - P49933, pig7 - P49934, pig8 - P49930, pig9 - P49931, pig10 - P49932, pig11 - P80054.

### Prediction and screening of ancestral tammar cathelicidin mature peptides

A mature peptide consensus sequence was generated using three prediction methods for ancestral sequence: PAML [36], GASP [37] and Ancescon [38]. Previously cloned and sequenced tammar cathelicidin sequences were used as input (ABV01941.1, ABV01938.1, ABV01939.1, ABV01940.1, ABV01942.1, ABV01943.1).

### Pouch microbe analysis

Pouch swabs were taken before birth (day 6 – 2), the day of birth (day 0) or one day after birth (day +1) from four wallabies by gently rubbing a sterile cotton swab over the mammary glands and around the bottom of the pouch. Swabs were then used to inoculate Nutrient Agar, Horse Blood Agar and MacConkey Agar. Plates were incubated aerobically for 24–48 hours at 37°C. Selected colonies were then identified by 16S rDNA sequencing. The closest genus and species match for each selected colony (%) are shown in Table 5

### Antimicrobial assays

Peptides were synthesized and HPLC purified (AusPep Pty Ltd). The two-stage radial diffusion assay used in this study has been described elsewhere [55]. Briefly, ∼4×10^6^ of mid-logarithmic-phase organisms were mixed into 10 ml of warm 0.8% agarose containing 0.03% (w/v) Trypticase soy broth (TSB) powder buffered with 10 mM sodium phosphate, pH 7.4. The synthetic peptides were serially diluted in 0.01% acetic acid and 5 µl peptide samples were loaded in 2.5 mm diameter wells. The plates were covered, and incubated at 37°C. After 3 hours, a 10 ml overlay gel composed of 6% TSB, 0.8% agarose in 10 mM sodium phosphate buffer (pH 7.4) was poured onto the plates. The plates were incubated overnight to allow the surviving organisms to form microcolonies. The clear zone was measured using a magnified transilluminator and expressed in units (1 mm  = 10 U) after subtracting the well diameter. The MIC is defined by the χ intercept of a regression line through zone diameters obtained from a series of serially diluted peptide samples. For multidrug-resistant bacteria, MICs were measured as described by micro-broth dilution in Mueller-Hinton broth (Oxoid, Hampshire, England) according to according to Clinical and Laboratory Standards Institute (*CLSI*) standards [56]. The MIC measurements were performed in duplicate and the data incorporated into a final MIC using standard methods. All the bacterial strains were tested on at least two different occasions. Mid-logarithmic phase cultures of *P. aeruginosa* 19056 muc were diluted in Mueller–Hinton broth to yield approximately 10^6^ cfu/ml. Peptides were added at the indicated concentrations and the suspensions were then incubated for different times in a shaking water bath at 35°C. At the end of each incubation time, the samples were serially diluted in buffered saline and plated onto nutrient agar with a spiral plater (Don Whitley Scientific, Australia). Colonies were counted after 17–22 h incubation at 35°C using an automatic colony counter (Don Whitley Scientific, Australia).
